# In silico discovery of antigenic proteins and epitopes of SARS-CoV-2 for the development of a vaccine or a diagnostic approach for COVID-19

**DOI:** 10.1038/s41598-020-79645-9

**Published:** 2020-12-28

**Authors:** Hüseyin Can, Ahmet Efe Köseoğlu, Sedef Erkunt Alak, Mervenur Güvendi, Mert Döşkaya, Muhammet Karakavuk, Adnan Yüksel Gürüz, Cemal Ün

**Affiliations:** 1 Department of Biology Molecular Biology Section, Faculty of Science, Ege University, Bornova, İzmir, Turkey; 2 Department of Parasitology, Faculty of Medicine, Ege University, Bornova, İzmir, Turkey; 3 Ödemiş Vocational School, Ege University, İzmir, Turkey

**Keywords:** Computational biology and bioinformatics, Immunology, Microbiology

## Abstract

In the genome of SARS-CoV-2, the 5′-terminus encodes a polyprotein, which is further cleaved into 15 non-structural proteins whereas the 3′ terminus encodes four structural proteins and eight accessory proteins. Among these 27 proteins, the present study aimed to discover likely antigenic proteins and epitopes to be used for the development of a vaccine or serodiagnostic assay using an in silico approach. For this purpose, after the full genome analysis of SARS-CoV-2 Wuhan isolate and variant proteins that are detected frequently, surface proteins including spike, envelope, and membrane proteins as well as proteins with signal peptide were determined as probable vaccine candidates whereas the remaining were considered as possible antigens to be used during the development of serodiagnostic assays. According to results obtained, among 27 proteins, 26 of them were predicted as probable antigen. In 26 proteins, spike protein was selected as the best vaccine candidate because of having a signal peptide, negative GRAVY value, one transmembrane helix, moderate aliphatic index, a big molecular weight, a long-estimated half-life, beta wrap motifs as well as having stable, soluble and non-allergic features. In addition, orf7a, orf8, and nsp-10 proteins with signal peptide were considered as potential vaccine candidates. Nucleocapsid protein and a highly antigenic GGDGKMKD epitope were identified as ideal antigens to be used in the development of serodiagnostic assays. Moreover, considering MHC-I alleles, highly antigenic KLNDLCFTNV and ITLCFTLKRK epitopes can be used to develop an epitope-based peptide vaccine.

## Introduction

Coronaviruses belonging to the family Coronaviridae and the order Nidovirales are a large family of enveloped positive-strand RNA viruses. Coronaviruses are zoonotic pathogens that infect both animals and humans, and may cause diseases in intestinal, liver, respiratory, and nervous systems. It has been stated that, among known coronaviruses, CoV-229E (alpha coronavirus), CoV-NL63 (alpha coronavirus), CoV-OC43 (beta coronavirus), CoV-HKU1 (beta coronavirus), severe acute respiratory syndrome coronavirus (SARS-CoV), Middle East respiratory syndrome coronavirus (MERS-CoV), and current SARS-CoV-2 can infect humans^1^.

In the genome of SARS-CoV-2, the 5′-terminus encodes a polyprotein (pp1ab), which is further cleaved into 15 non-structural proteins (nsp-1 to nsp-10 and nsp-12 to nsp-16) whereas the 3′ terminus encodes four structural proteins including spike (S), envelope (E), membrane (M), and nucleocapsid (N) proteins and eight accessory proteins (3a, 3b, p6, 7a, 7b, 8b, 9b, and orf14)^1^. Comparative genomic analyses have revealed that SARS-CoV-2 shared more nucleotide homology with SARS-CoV than MERS-CoV^2,3^. Also, it has been reported that RBD of SARS-CoV-2 binds to the human ACE2 receptor in a higher affinity than that of SARS-CoV as well as the presence of an insertion in the polybasic cleavage region in SARS-CoV-2 may increase the infectivity of the virus^4–6^. The novel coronavirus, SARS-CoV-2 causing COVID-19 has been first reported in Wuhan City of Hubei Province in China in December 2019, and then, COVID-19 has spread from China to 211 different countries with more than 28 million cases including more than 900 thousand deaths in record time^7^.

Since it has been known that no specific therapeutic agents that target SARS-CoV-2 are currently available, the development of an urgent vaccine against SARS-CoV-2 is inevitable. In vaccine development, traditional or recombinant vaccine methods are being used. Traditional approaches, based on inactivated or live attenuated viruses, can be applied for vaccine development but it has been reported that these approaches have some limitations such as being time-consuming, having problems in the production of non-abundant proteins and pathogens^8^. This condition prevents the development of new vaccines against pathogens causing the outbreaks leading to a pandemic. On the other hand, to overcome these problems, new recombinant vaccine development strategies allowing several genes obtained from different pathogenic agents to be cloned, expressed, and purified to be used as vaccine candidates are applied^9^. During new recombinant vaccine design, reverse vaccinology (RV) in silico approach provides detailed preliminary prediction about vaccine candidates by using genome sequences that can be ultimately translated into proteins. Utilisation of RV in silico approach is rather crucial because of offering a prediction for the antigenicity, the epitope regions of B and T cells as well as other parameters such as signal peptide, subcellular localisation, and solubility about targeted proteins^8,10^. Currently, docking analysis demonstrating the binding among predicted epitopes and selected alleles of MHC-I and MHC-II became an important part of RV in silico approach^8^. Result obtained from in silico prediction has utmost importance for preventing the failures that can be encountered at the end of wet lab studies or even late stages of clinical trials.

In this context, the present study aimed to analyse the full genome of SARS-CoV-2 (reference isolate Wuhan-Hu-1; Accession number: NC_045512.2) in order to discover likely antigenic proteins and epitopes to be used for the development of a vaccine candidate or serodiagnostic assay using an RV in silico approach as previously described^8,11–13^. For this purpose, surface proteins including S, E, and M proteins as well as proteins that were predicted to have a signal peptide were identified as probable vaccine candidates whereas the remaining were considered as probable antigens to be used in the development of a serodiagnostic assay. During in silico analyses, physico-chemical parameters, secondary structure, subcellular localisation, transmembrane helices, antigenicity, and signal peptide were predicted for 27 proteins of the reference Wuhan genome. Later, for structural proteins as well as proteins that have a signal peptide, allergenicity, BetaWrap motifs, similarity with host proteins, post-translational modifications (PTMs), and B/T cell epitopes were predicted and selected epitopes were docked with receptors of MHC-I/II alleles. Finally, effects of variations frequently occurred in structural proteins and the proteins that were predicted to have signal peptide on antigenicity, signal peptide, solubility, BetaWrap motifs, PTMs and epitope regions were investigated.

## Results

### Physico-chemical parameters

The number of amino acids varied from 75 to 1273 among structural proteins. The largest one was S protein with ~ 142 kDa whereas E protein with ~ 8.4 kDa was the smallest one (Table 1). Among non-structural proteins, except orf1ab, the number of amino acids varied from 43 to 275. Orf3a with ~ 32 kDa was the largest protein whereas orf7b with 5.2 kDa was the smallest protein (Table 1). Each non-structural protein that is encoded by orf1ab was also analysed, and the number of amino acids was detected to vary from 83 to 1945. Nsp-3 with ~ 218 kDa molecular weight was one of the largest proteins whereas the smallest one was nsp-7 with ~ 9.3 kDa size (Table 1). When all proteins encoded by the full genome were analysed, the theoretical PI value was between 4.6 and 10.07. Among structural proteins, only S protein was negatively charged whereas E, M, and N protein were positively charged. In addition, orf7a, orf10, nsp-6, nsp-9, nsp-13, nsp-14, and nsp-16 proteins were positively charged whereas the remaining proteins were negatively charged except nsp-4 and nsp-8 that were neutral. The estimated half-life was 30 h for all proteins, except proteins that were encoded by orf1ab. Only nsp-1 in orf1ab had 30 h estimated half-life. According to the instability index, N protein was found as instable while S, E, M structural proteins and most of the non-structural proteins were found as stable. The aliphatic index showed a significant variation ranging between 52.53 to 144 among all proteins. The grand average of hydropathicity value was found negative in S and N proteins as well as in most of the non-structural proteins that were encoded by orf1ab (Table 1).

**Table 1:** Physico-chemical parameter results predicted by ExPASyProtParam.

	Protein name	Number of amino acids	Molecular weight	Theoretical PI	Total number of negatively charged residues (Asp + Glu)	Total number of positively charged residues (Arg + Lys)	The estimated half-life (hour)	The instability index (II)	Aliphatic index	GRAVY^a^
Structural proteins	S protein	1273	141,178.47	6.24	110	103	30	33.01/stable	84.67	− 0.079
	E protein	75	8365.04	8.57	3	5	30	38.68/stable	144.00	1.128
	M protein	222	25,146.62	9.51	13	21	30	39.14/stable	120.86	0.446
	N protein	419	45,625.70	10.07	36	60	30	55.09/unstable	52.53	− 0.971
Non-structural proteins	orf3a	275	31,122.94	5.55	24	17	30	32.96/stable	103.42	0.275
	orf6	61	7272.54	4.60	9	5	30	31.16/stable	130.98	0.233
	orf7a	121	13,744.17	8.23	10	12	30	48.66/unstable	100.74	0.318
	orf7b	43	5180.27	4.17	5	0	30	50.96/unstable	156.51	1.449
	orf8	121	13,831.01	5.42	13	9	30	45.79/unstable	97.36	0.219
	orf10	38	4449.23	7.93	1	2	30	16.06/stable	107.63	0.637
Pp1ab	nsp-1	180	19,775.31	5.36	27	19	30	28.83/stable	89.72	− 0.378
	nsp-2	638	70,511.38	6.25	74	70	4.4	36.06/stable	88.93	− 0.062
	nsp-3	1945	217,252.61	5.56	222	185	4.4	36.56/stable	86.22	− 0.175
	nsp-4	500	56,183.98	7.16	37	37	1.3	34.09/stable	95.50	0.343
	nsp-5	306	33,796.64	5.95	26	22	1.9	27.65/stable	82.12	− 0.019
	nsp-6	290	33,033.69	9.11	11	21	1.9	22.94/stable	111.55	0.790
	nsp-7	83	9239.82	5.18	10	8	1.9	51.97/unstable	117.35	0.199
	nsp-8	198	21,881.08	6.58	22	22	4.4	37.78/stable	88.33	− 0.192
	nsp-9	113	12,378.20	9.10	9	13	1.4	34.17/stable	82.92	− 0.227
	nsp-10	139	14,789.92	6.29	11	10	4.4	34.56/stable	61.80	− 0.068
	nsp-12	932	106,660.24	6.14	106	94	1.9	28.32/stable	78.43	− 0.224
	nsp-13	601	66,854.75	8.66	52	64	4.4	33.31/stable	84.49	− 0.096
	nsp-14	527	59,815.67	7.80	50	52	4.4	28.85/stable	78.96	− 0.134
	nsp-15	346	38,813.40	5.06	46	34	1.9	36.28/stable	95.09	− 0.076
	nsp-16	298	33,323.32	7.59	26	27	1.9	26.11/stable	90.64	− 0.086

^a^GRAVY means the Grand average of hydropathicity.

### Secondary structure

According to results obtained from structural proteins, the alpha helix was between ~ 22 and 47%, that of the extended strand was between ~ 10 and 22%, and that of the random coil was between ~ 40 and 60%. For non-structural proteins, the alpha helix varied between 0 and 69%, that of the extended strand varied between ~ 3 and 47%, and that of the random coil varied between ~ 28 and 58% (Table 2).

**Table 2:** Secondary structures results predicted by GOR IV.

	Protein name	Alpha helix (%)	Extended strand (%)	Random coil (%)
Structural proteins	S protein	21.52	22.07	56.40
	E protein	33.33	13.33	53.33
	M protein	46.85	13.51	39.64
	N protein	30.55	9.79	59.67
Non-structural proteins	orf3a	18.91	32.73	48.36
	orf6	50.82	3.28	45.90
	orf7a	23.97	26.45	49.59
	orf7b	55.81	6.98	37.21
	orf8	0.00	46.28	53.72
	orf10	0.00	44.74	55.26
Pp1ab	nsp-1	26.11	15.56	58.33
	nsp-2	26.33	18.97	54.70
	nsp-3	26.12	24.27	49.61
	nsp-4	19.00	28.40	52.60
	nsp-5	14.05	37.58	48.37
	nsp-6	28.28	34.48	37.24
	nsp-7	68.67	3.61	27.71
	nsp-8	64.14	7.07	28.79
	nsp-9	30.97	19.47	49.56
	nsp-10	11.51	34.53	53.96
	nsp-12	27.15	23.50	49.36
	nsp-13	17.64	26.29	56.07
	nsp-14	13.85	29.79	56.36
	nsp-15	18.79	26.59	54.62
	nsp-16	14.43	33.22	52.35

### Antigenicity

All structural proteins were predicted as probable antigen. Antigenicity value varied from 0.4661 to 0.6025. E protein had the highest antigenicity value whereas S protein had the lowest antigenicity value. Antigenicity values did not dramatically change among the original Wuhan and variant proteins. Interestingly, all non-structural proteins were also predicted as probable antigen, except nsp-16 encoded by orf1ab. In addition, orf7b had the highest antigenicity value with 0.8462 among all proteins. According to Wuhan orf8 antigenicity value, variant V62L had a higher antigenicity value whereas variant L84S had a lower antigenicity value (Table 3).

**Table 3:** Solubility, transmembrane helices, localisation and antigenicity results predicted by SolPro, TMHMM, Virus-mPLoc and Vaxijen, respectively.

	Proteins	SolPro	TMHMM	Virus-mPLoc	Vaxijen v2.0 value
Structural proteins	S protein	Soluble	1	Host endoplasmic reticulum	0.4661 (probable antigen)
	E protein	Soluble	1	Host endoplasmic reticulum	0.6025 (probable antigen)
	M protein	Insoluble	3	Host cell membrane, endoplasmic reticulum	0.5102 (probable antigen)
	N protein	Soluble	0	Host cell membrane	0.5059 (probable antigen)
Non-structural proteins	orf3a	Insoluble	3	Host cell membrane	0.4945 (probable antigen)
	orf6	Soluble	0	Host endoplasmic reticulum	0.6131 (probable antigen)
	orf7a	Soluble	1	Host endoplasmic reticulum	0.6441 (probable antigen)
	orf7b	Soluble	1	^a^	0.8462 (probable antigen)
	orf8	Soluble	0	Host cell membrane, endoplasmic reticulum, cytoplasm	0.6502 (probable antigen)
	orf10	Soluble	0	^a^	0.7185 (probable antigen)
Pp1ab	nsp-1	Soluble	0	Host cytoplasm	0.4064 (probable antigen)
	nsp-2	Insoluble	0	Host cytoplasm	0.4034 (Probable antigen)
	nsp-3	^a^	4	Host cytoplasm	0.5142 (probable antigen)
	nsp-4	Insoluble	4	Host cell membrane, endoplasmic reticulum	0.4691 (probable antigen)
	nsp-5	Insoluble	0	Host cell membrane	0.4159 (probable antigen)
	nsp-6	Soluble	8	Host cell membrane	0.5813 (probable antigen)
	nsp-7	Insoluble	0	Host cytoplasm	0.4167 (probable antigen)
	nsp-8	Soluble	0	Host cytoplasm	0.4008 (probable antigen)
	nsp-9	Soluble	0	Host cell membrane, cytoplasm	0.6476 (Probable antigen)
	nsp-10	Insoluble	0	Host cell membrane, endoplasmic reticulum	0.4039 (probable antigen)
	nsp-12	Insoluble	0	Host cytoplasm	0.4064 (probable antigen)
	nsp-13	Insoluble	0	Host nucleus	0.4480 (probable antigen)
	nsp-14	Insoluble	0	Host cytoplasm, nucleus	0.4138 (probable antigen)
	nsp-15	Insoluble	0	Host cytoplasm	0.5554 (probable antigen)
	nsp-16	Insoluble	0	Host cytoplasm	0.3800 (probable non-antigen)

^a^Could not be retrieved.

### Solubility

According to solubility prediction, S, E, and N proteins were soluble. Among non-structural proteins, orf3a as well as nsp-2, 4, 5, 7, 10, 12, 13, 14, 15, and 16 proteins encoded by orf1ab were predicted as insoluble whereas the remaining orf6 to orf10 and nsp-1, 6, 8, 9 were predicted as soluble. The solubility prediction of another protein, nsp-3 encoded by orf1ab, could not be retrieved due to large fragment size (Table 3).

### Subcellular localisation and transmembrane helices

The number of transmembrane helices varied from 0 to 3 among structural proteins. The number of transmembrane helices was the lowest in N protein whereas it was the highest in M protein. Among non-structural proteins, although the number of transmembrane helices varied from 0 to 8, most of them had no transmembrane helices (Table 3). When subcellular localisation predictions were examined, S, M, and N proteins were predicted to be in the host endoplasmic reticulum. E as well as M proteins were also predicted to be in the host cell membrane. Non-structural proteins were predicted to locate in cell membrane, endoplasmic reticulum, cytoplasm, nucleus (Table 3).

### Signal peptide

According to the prediction of signal peptide based on four different parameters, only S protein and its variant were predicted to have a signal peptide during the analyses of structural proteins. Among non-structural proteins, orf7a, orf8, variant of orf8, and nsp-10 were predicted to have a signal peptide (Table 4). Any variations did not change the results of signal peptide.

**Table 4:** The signal peptide results predicted by Signal-BLAST.

	Protein name	Sensitivity	Specificity	Balanced prediction	Cleavage site
Structural proteins	**S protein**	Yes	Yes	Yes	Yes
	**Variant D614G**	Yes	Yes	Yes	Yes
	E protein	No	No	No	Yes
	Variant L37H	No	No	No	No
	M protein	No	No	No	No
	Variant M T175M	No	No	No	No
	N protein	No	No	No	Yes
	Variant N P13L	No	No	No	Yes
	Variant N S194L	No	No	No	Yes
	Variant NS197L	No	No	No	Yes
	Variant N R203K/G204R	No	No	No	Yes
Non-structural proteins	orf3a	No	No	No	No
	orf6	No	No	No	Yes
	**orf7a**	Yes	Yes	Yes	Yes
	ORF7b	No	No	No	Yes
	**orf8**	Yes	Yes	Yes	Yes
	**Variant orf8 S24L**	Yes	Yes	Yes	Yes
	**Variant orf8 V62L**	Yes	Yes	Yes	Yes
	**Variant orf8 L84S**	Yes	Yes	Yes	Yes
	orf10	No	No	No	Yes
Pp1ab	nsp-1	No	No	No	Yes
	nsp-2	No	No	No	Yes
	nsp-3	No	No	No	No
	nsp-4	No	No	No	Yes
	nsp-5	Yes	No	Yes	Yes
	nsp-6	Yes	No	Yes	Yes
	nsp-7	No	No	No	Yes
	nsp-8	No	No	No	No
	nsp-9	Yes	No	Yes	Yes
	**nsp-10**	Yes	Yes	Yes	Yes
	nsp-12	No	No	No	Yes
	nsp-13	No	No	No	No
	nsp-14	No	No	No	No
	nsp-15	No	No	No	No
	nsp-16	No	No	No	No

Bold indicates proteins that have a signal peptide.

### Allergenicity

None of the proteins including variant proteins showed allergenic properties for MEME/MAST motif and IgE epitopes (Table 5).

**Table 5:** Allergenicity, BetaWrap motifs and host proteome similarity results predicted by AlgPred, BetaWrap and BlastP, respectively.

	Proteins	AlgPred		BetaWrap motifs	BlastP
		IgE epitopes	MEME/MAST motif		
Structural proteins	S protein Variant S (D614G)	–	Non allergen	P value: 0.014	No
	E protein Variant E (L37H)	–	Non allergen	No	No
	M protein Variant M (T175M)	–	Non allergen	No	No
	N protein Variant N (P13L) Variant N (S194L) Variant N (S197L) Variant N (R203K 204R)	–	Non allergen	No	No
Non-structural proteins	orf7a	–	Non allergen	No	No
	orf8 Variant orf8 (S24L) Variant orf8 (V62L) Variant orf8 (L84S)	–	Non allergen	No	No
Pp1ab	nsp-10	–	Non allergen	No	No

### BetaWrap motifs

Among all proteins analysed, only S protein and its variant (D614G) were predicted to contain BetaWrap motifs (Table 5).

### Similarity with host proteome

No significant similarity was predicted between analysed viral proteins including variant proteins and host proteins (Table 5).

### B cell epitopes

A lot of linear B cell epitopes were predicted for S, variant S (D614G), N and variants of N (P13L, S194L, S197L, and R203K/G204R), E, and variant E (L37H), orf8 and variants of orf8 (S24L, V62L and L84S) and nsp-10 proteins using Bcepred and IEDB. Epitopes that were predicted in both Bcepred and IEDB, and detected as probable antigen were presented in Table 6. Obtained predictions showed that nearly all epitopes had more antigenicity value than those of their own proteins. Interestingly, an epitope (VDEAGSKS) corresponding to variant orf8 (S24L) was predicted non-antigen because of adding valine amino acid to lead of epitope as different from the original Wuhan sequence. Variant N (P13L) had a specific epitope (AEGSRGGSQASSRSSSRSRNS) with a high antigenicity value that was not predicted for N or other variant N proteins. Among these analysed proteins, the highest antigenicity value (1.4530) was predicted for an epitope (GGDGKMKD) belonging to N protein and its variants. Another epitope (THTGTGQ) that had a high antigenicity value of 1.0789 was predicted in Nsp-10 encoded by orf1ab. Also, any antigenic epitope was not predicted for M and orf7a proteins. All predicted probable antigenic epitopes were depicted in Table 6.

**Table 6:** B cell epitopes predicted by both Bcepred and IEDB and antigenicity value predicted by Vaxijen v2.0.

	Proteins	Antigenicity value	B cell epitopes	Antigenicity value for epitopes
Vaccine candidate epitopes^a^	S protein	0.4661 (probable antigen)	VYYHKNNKSW YAWNRKRISN GDEVRQ NLDSKV	0.4497 (probable antigen) 0.5855 (probable antigen) 0.6701 (probable antigen) 0.7443 (probable antigen)
	Variant (D614G)	0.4638 (probable antigen)		
	E protein	0.6025 (probable antigen)	SRVKNLNSSRVP	0.5572 (probable antigen)
	Variant E (L37H)	0.6298 (probable antigen)		
	M protein	0.5102 (probable antigen)	ND	–
	Variant M (T175M)	0.4990 (probable antigen)		
	orf8	0.6502 (probable antigen)	DEAGSKS	0.5885 (probable antigen)
	Variant orf8 (V62L)	0.6734 (probable antigen)		
	Variant orf8 (L84S)	0.6063 (probable antigen)		
	Variant orf8 (S24L)	0.6581 (probable antigen)	VDEAGSKS	0.2132 (probable NON-antigen)
	nsp-10	0.4039 (probable antigen)	THTGTGQ	1.0789 (probable antigen)
Diagnostic epitopes	N protein	0.5059 (probable antigen)	NGPQNQRNAP NTNSSPDDQI GGDGKMKD AEGSRGGSQASSRSSSRSRNSSRNS AGNGGD ESKMSGKGQQQQGQT PQRQKKQQT QSMSSADS	0.5058 (probable antigen) 0.4913 (probable antigen) 1.4530 (probable antigen) 0.8682 (probable antigen) 0.8201 (probable antigen) 0.8163 (probable antigen) 0.5997 (probable antigen) 0.4864 (probable antigen)
	Variant N (P13L)	0.5119 (probable antigen)		
	Variant N (S194L	0.4989 (probable antigen)		
	Variant N (S197L)	0.5139 (probable antigen)		
	Variant N (R203K/G204R)	0.5068 (probable antigen)		
	Variant N (P13L)	0.5119 (probable antigen)	AEGSRGGSQASSRSSSRSRNS	0.9061 (probable antigen)

*ND* not detected.

^a^Vaccine candidate epitopes can also be used to develop serodiagnostic assays.

### MHC-I and MHC-II epitopes

A lot of MHC-I epitopes were predicted as probable antigen (Table 7). Antigenicity values belonging to epitopes were generally predicted higher than those of their own proteins. Among structural proteins, an epitope (KLNDLCFTNV) that had the highest antigenicity value (2.6927) was predicted in S protein and its variant (D614G). For non-structural proteins, an epitope (ITLCFTLKRK) in orf7a had the highest antigenicity value (2.5150). Any antigenic epitope was not predicted for nsp-10. On the other hand, KWPWYIWLGF, FLAFVVFLLV, FARTRSMWSF, and RNRFLYIIKL, AQFAPSASAF and LGIITTVAAF epitopes belonging to S (including variant D614G), E (including variant L37H), M (including variant T175M), N (including variants P13L, S194L, S197L, and R203K/G204R) and orf8 (including S24L, V62L, and L84S), respectively, had an IC50 value lower than 10 and a percentile rank varying from 0.02 from 0.1, indicating a strong binding among the epitope and MHC-I alleles. Also, T175M and S194L variations in M and N proteins caused the prediction of additional epitopes that are specific to own themselves.

**Table 7:** Epitopes specific to selected MHC-I alleles.

	Proteins	Allele	Start	End	Peptide	IC50	Percentile rank	Antigenicity
Vaccine candidate epitopes^a^	S protein Variant S (D614G)	HLA-A*24:02	1211	1220	KWPWYIWLGF	9.04	0.02	1.3904 (probable antigen)
		HLA-B*15:01	754	763	LQYGSFCTQL	13.44	0.06	1.4443 (probable antigen)
		HLA-A*02:01	386	395	KLNDLCFTNV	15.27	0.14	2.6927 (probable antigen)
		HLA-B*07:02	680	689	SPRRARSVAS	18.4	0.07	0.5591 (probable antigen)
		HLA-A*02:01	1209	1218	YIKWPWYIWL	18.81	0.2	0.8847 (probable antigen)
		HLA-A*02:01	515	524	FELLHAPATV	20.98	0.23	0.5982 (probable antigen)
		HLA-A*24:02	488	497	CYFPLQSYGF	27.59	0.04	0.7776 (probable antigen)
		HLA-B*15:01	49	58	HSTQDLFLPF	27.79	0.17	0.5162 (probable antigen)
		HLA-A*02:01	2	11	FVFLVLLPLV	32.64	0.37	0.8044 (probable antigen)
		HLA-A*02:01	268	277	GYLQPRTFLL	36.12	0.4	0.7535 (probable antigen)
		HLA-B*15:01	698	707	SLGAENSVAY	37.99	0.22	0.6175 (probable antigen)
		HLA-A*03:01	408	417	RQIAPGQTGK	38.0	0.15	1.7893 (probable antigen)
		HLA-A*03:01	1064	1073	HVTYVPAQEK	38.88	0.15	1.0786 (probable antigen)
		HLA-A*24:02	897	906	PFAMQMAYRF	38.93	0.05	1.1051 (probable antigen)
		HLA-A*03:01	724	733	TEILPVSMTK	42.81	0.17	1.4160 (probable antigen)
		HLA-A*24:02	1207	1216	EQYIKWPWYI	45.33	0.06	1.1122 (probable antigen)
	E protein	HLA-A*02:01	20	29	FLAFVVFLLV	9.95	0.1	0.5651 (probable antigen)
	Variant E (L37H)	HLA-A*02:01	18	27	LLFLAFVVFL	32.72	0.37	0.6159 (probable antigen)
	orf7a	HLA-A*02:01	101	110	FLIVAAIVFI	13.43	0.13	0.6283 (probable antigen)
		HLA-B*40:01	40	49	YEGNSPFHPL	18.20	0.06	0.6193 (probable antigen)
		HLA-A*03:01	76	85	QLRARSVSPK	28.96	0.12	1.4738 (probable antigen)
		HLA-B*15:01	56	65	LTCFSTQFAF	29.06	0.18	1.1543 (probable antigen)
		HLA-A*03:01	23	32	CVRGTTVLLK	36.79	0.15	0.7426 (probable antigen)
		HLA-B*07:02	47	56	HPLADNKFAL	39.32	0.16	0.6385 (probable antigen)
		HLA-A*03:01	110	119	ITLCFTLKRK	39.65	0.15	2.5150 (probable antigen)
	orf8	HLA-B*15:01	7	16	LGIITTVAAF	6.51	0.02	0.7595 (probable antigen)
	Variant orf8 (S24L) Variant orf8 (V62L) Variant orf8 (L84S)	HLA-B*40:01	109	118	LEYHDVRVVL	41.00	0.12	0.9885 (probable antigen)
	M protein Variant M (T175M)	HLA-B*08:01	103	112	FARTRSMWSF	7.87	0.02	0.9202 (probable antigen)
		HLA-B*27:05	42	51	RNRFLYIIKL	8.00	0.02	0.7016 (probable antigen)
		HLA-A*02:01	51	60	LIFLWLLWPV	10.14	0.1	0.5633 (probable antigen)
		HLA-A*02:01	26	35	FLFLTWICLL	10.42	0.1	1.1459 (probable antigen)
		HLA-B*15:01	56	65	LLWPVTLACF	11.12	0.05	0.9864 (probable antigen)
		HLA-B*27:05	71	80	YRINWITGGI	11.15	0.02	1.3250 (probable antigen)
		HLA-B*15:01	17	26	LEQWNLVIGF	14.47	0.07	1.0564 (probable antigen)
		HLA-A*02:01	53	62	FLWLLWPVTL	16.02	0.15	0.9162 (probable antigen)
		HLA-A*02:01	88	97	VGLMWLSYFI	17.08	0.18	0.6741 (probable antigen)
		HLA-B*40:01	136	145	SELVIGAVIL	17.83	0.06	0.6521 (probable antigen)
		HLA-A*02:01	61	70	TLACFVLAAV	20.28	0.22	1.2318 (probable antigen)
		HLA-A*03:01	171	180	ATSRTLSYYK	21.49	0.08	0.4317 (probable antigen)
		HLA-B*27:05	100	109	FRLFARTRSM	31.60	0.1	0.4609 (probable antigen)
		HLA-B*15:01	169	178	TVATSRTLSY	40.92	0.23	0.8259 (probable antigen)
		HLA-B*58:01	22	31	LVIGFLFLTW	46.78	0.22	1.3639 (probable antigen)
	Variant M (T175M)	HLA-A*02:01	89	98	GLMWLSYFIA	32.32	0.29	0.2752 (probable antigen)
		HLA-A*02:01	45	54	FLYIIKLIFL	36.01	0.32	0.5871 (probable antigen)
Diagnostic epitopes	N protein Variant N (P13L) Variant N (S194L Variant N (S197L) R203K/G204R	HLA-B*15:01	305	314	AQFAPSASAF	3.58	0.02	0.5986 (probable antigen)
		HLA-A*03:01	361	370	KTFPPTEPKK	11.43	0.02	0.7657 (probable antigen)
		HLA-B*40:01	322	331	MEVTPSGTWL	28.62	0.09	0.6342 (probable antigen)
		HLA-A*01:01	78	87	SSPDDQIGYY	33.46	0.1	0.4533 (probable antigen)
		HLA-B*07:02	66	75	FPRGQGVPIN	33.85	0.14	0.7135 (probable antigen)
		HLA-A*02:01	315	324	FGMSRIGMEV	36.81	0.4	0.8800 (probable antigen)
		HLA-B*08:01	104	113	LSPRWYFYYL	37.51	0.11	1.3486 (probable antigen)
	Variant N (S194L)	HLA-B*15:01	8	17	NQRNALRITF	37.01	0.2	1.1293 (probable antigen)

^a^Vaccine candidate epitopes can also be used to develop serodiagnostic assays.

Similarly, a lot of MHC-II epitopes were predicted as probable antigen (Table 8). Also, nearly all epitopes had higher antigenicity values than those of their own proteins. Among structural proteins, PTNFTISVTTEILPV and VTLAILTAHRLCAYC epitopes predicted in S protein (including variant D614G) and variant L37H had the highest antigenicity value. For non-structural proteins, orf7a had an epitope (IVFITLCFTLKRKTE) that was predicted as a probable antigen with a high antigenicity value (1.8597). Any antigenic epitope was not predicted for nsp-10. Among MHC-II epitopes, although there were a lot of epitopes with low percentile rank, only two epitopes (SKWYIRVGARKSAPL and KWYIRVGARKSAPLI) that had an IC50 value lower than 10, indicating a strong binding among epitope and MHC-II alleles, was detected in orf8 and its variants. In addition, variant M protein (T175M) and orf8 variants (S24L and V62L) had specific epitopes with high antigenicity values as different from original Wuhan M and orf8 proteins.

**Table 8:** Epitopes specific to selected MHC-II alleles.

	Proteins	Allele	Start	End	Peptide	IC50	Percentile rank	Antigenicity
Vaccine candidate epitopes^a^	S protein	HLA-DRB3*02:02	115	129	QSLLIVNNATNVVIK	13.08	0.02	0.4343 (probable antigen)
	D614G	HLA-DRB5*01:01	894	908	LQIPFAMQMAYRFNG	20.96	0.59	0.7205 (probable antigen
		HLA-DRB1*07:01	715	729	PTNFTISVTTEILPV	25.32	0.63	1.1349 (probable antigen)
		HLA-DRB3*02:02	1091	1105	REGVFVSNGTHWFVT	25.75	0.2	0.4461 (probable antigen)
		HLA-DRB1*07:01	691	705	SIIAYTMSLGAENSV	35.71	1.4	0.5691 (probable antigen)
		HLA-DRB1*07:01	199	213	GYFKIYSKHTPINLV	40.49	1.9	0.9278 (Probable antigen)
		HLA-DRB1*15:01	52	66	QDLFLPFFSNVTWFH	44.58	0.77	0.4159 (probable antigen)
	Variant E (L37H)	HLA-DRB5*01:01	29	43	VTLAILTAHRLCAYC	33.95	1.90	1.0545 (probable antigen)
	M protein	HLA-DRB5*01:01	98	112	ASFRLFARTRSMWSF	16.26	0.29	0.7304 (probable antigen)
	Variant M (T175M)	HLA-DRB5*01:01	175	189	TLSYYKLGASQRVAG	16.67	0.30	0.4376 (probable antigen)
		HLA-DRB5*01:01	34	48	LLQFAYANRNRFLYI	26.30	1.10	0.7387 (Probable antigen)
		HLA-DRB1*07:01	174	188	RTLSYYKLGASQRVA	31.50	1.10	0.5644 (probable antigen)
		HLA-DRB1*07:01	165	179	PKEITVATSRTLSYY	37.66	1.60	0.7003 (probable antigen)
		HLA-DRB5*01:01	31	45	WICLLQFAYANRNRF	44.95	3.10	0.6994 (probable antigen)
		HLA-DRB3*02:02	34	48	LLQFAYANRNRFLYI	46.63	0.75	0.7387 (probable antigen)
		HLA-DRB1*15:01	32	46	ICLLQFAYANRNRFL	47.01	0.82	0.6221 (Probable antigen)
		HLA-DRB5*01:01	139	153	VIGAVILRGHLRIAG	48.64	3.40	0.4903 (probable antigen)
	Variant M (T175M)	HLA-DRB5*01:01	100	114	FRLFARTRSMWSFNP	28.34	1.20	0.8873 (probable antigen)
		HLA-DRB5*01:01	35	49	LQFAYANRNRFLYIL	36.31	2.10	0.9542 (probable antigen)
		HLA-DRB5*01:01	138	152	LVIGAVILRGHLRIA	48.98	3.40	0.8769 (probable antigen)
	orf7a	HLA-DRB5*01:01	71	85	VKHVYQLRARSVSPK	40.51	2.6	1.0865 (Probable antigen)
		HLA-DRB5*01:01	107	121	IVFITLCFTLKRKTE	49.35	3.4	1.8597 (probable antigen)
	orf8 Variant orf8 (S24L) Variant orf8 (V62L) Variant orf8 (L84S) Variant orf8 (S24L) Variant orf8 (V62L)	HLA-DRB5*01:01	43	57	SKWYIRVGARKSAPL	8.77	0.05	0.8829 (probable antigen)
		HLA-DRB3*01:01	28	42	HQPYVVDDPCPIHFY	19.07	0.06	0.5587 (probable antigen)
		HLA-DRB5*01:01	44	58	KWYIRVGARKSAPLI	9.66	0.07	0.9009 (probable antigen)
		HLA-DRB5*01:01	45	59	WYIRVGARKSAPLIE	15.09	0.27	1.0427 (probable antigen)
		HLA-DRB3*01:01	27	41	QHQPYVVDDPCPIHF	21.85	0.07	0.8637 (probable antigen)
		HLA-DRB5*01:01	39	53	IHFYSKWYIRVGARK	23.64	0.77	1.0268 (probable antigen)
		HLA-DRB5*01:01	46	60	YIRVGARKSAPLIEL	40.62	2.60	0.9809 (probable antigen)
Diagnostic epitopes	N protein Variant N (P13L) Variant N (S194L Variant N (S197L) Variant N (R203K/G204R) Variant N (P13L) Variant N (S197L)	HLA-DRB5*01:01	264	278	ATKAYNVTQAFGRRG	29.21	1.40	0.7146 (probable antigen)
		HLA-DRB1*07:01	303	317	QIAQFAPSASAFFGM	32.18	1.10	0.4032 (probable antigen)
		HLA-DRB5*01:01	84	98	IGYYRRATRRIRGGD	15.45	0.27	0.6649 (probable antigen)
		HLA-DRB1*07:01	328	342	GTWLTYTGAIKLDDK	24.62	0.58	0.9934 (probable antigen)
		HLA-DRB1*07:01	305	319	AQFAPSASAFFGMSR	41.34	2	0.5266 (probable antigen)

^a^Vaccine candidate epitopes can also be used to develop serodiagnostic assays.

### Post-translational modifications

S protein and its variant (D614G) were predicted to have highly N-glycosylated and phosphorylated sites as well as a few O-glycosylated and acetylated sites. M (including T175M), E (including L37H), orf7a, and nsp10 proteins were predicted to have N-glycosylated and phosphorylated sites while orf7a was predicted to have an acetylation site. Orf8 and its variants were predicted to have N-glycosylated and phosphorylated sites whereas two additional phosphorylation sites, one of which locate in the exposed surface and the other one is buried, were predicted in only variant L84S. In addition, N protein and its four variants were predicted to have N-/O-glycosylated, phosphorylated and acetylated sites. When N protein and its variant were compared, the number of O-glycosylation, acetylation, or phosphorylation sites showed minor alterations. In addition, post-translational modifications within significant epitopes were shown in Table 9.

**Table 9:** Additional analysis performed for significant epitopes.

Epitopes	N glycosylation region	Acetylation region	Phosphorylation region	Solvent-exposure amino acids	Lineages
THTGTGQ	–	–	–	**THTGT**GQ	All lineages
GGDGKMKD	–	GGDG**K**M**K**D	–	GGDG**KMKD**	All lineages
KLNDLCFTNV	KLNDLCFTNV	**K**LNDLCFTNV	–	KLNDLCFTNV	All lineages
ITLCFTLKRK	–	ITLCFTL**K**R**K**	–	**IT**LCFTLKRK	All lineages
PTNFTISVTTEILPV	PT**N**FTISVTTEILPV	–	PTNFTI**S**VTTEILPV	PTNF**T**ISVTTEILPV	All lineages
IVFITLCFTLKRKTE	–	IVFITLCFTL**K**R**K**TE	IVFITLCF**T**LKRKTE	**IVFIT**LCFTLKRKTE	All lineages
KWPWYIWLGF	–	**K**WPWYIWLGF	–	**KWPWYIWLGF**	All lineages
FLAFVVFLLV	–	–	–	**FLAFVVFLLV**	All lineages
LGIITTVAAF	–	–	–	LGIITTVAAF	All lineages
FARTRSMWSF	–	–	–	FART**RSMW**S**F**	All lineages
RNRFLYIIKL	RNRFLYIIKL	RNRFLYII**K**L	–	**RNRFLYII**KL	All lineages
SKWYIRVGARKSAPL	–	S**K**WYIRVGAR**K**SAPL	SKW**Y**IRVGARK**S**APL	SKWYIRVGA**RKSAPL**	All lineages
KWYIRVGARKSAPLI	–	**K**WYIRVGAR**K**SAPLI	KW**Y**IRVGARK**S**APLI	KWYIRVGA**RKSAPLI**	All lineages

Only underlined amino acid shows the moderate solvent exposure intensity.

Both underlined and bold amino acids show the high solvent exposure intensity.

Analysed common lineages are A, A.1, A.2, A.3, A.5, B, B.1, B.1.1, B.2, B.3, B.4.

### Docking analysis

**Figure 1:**
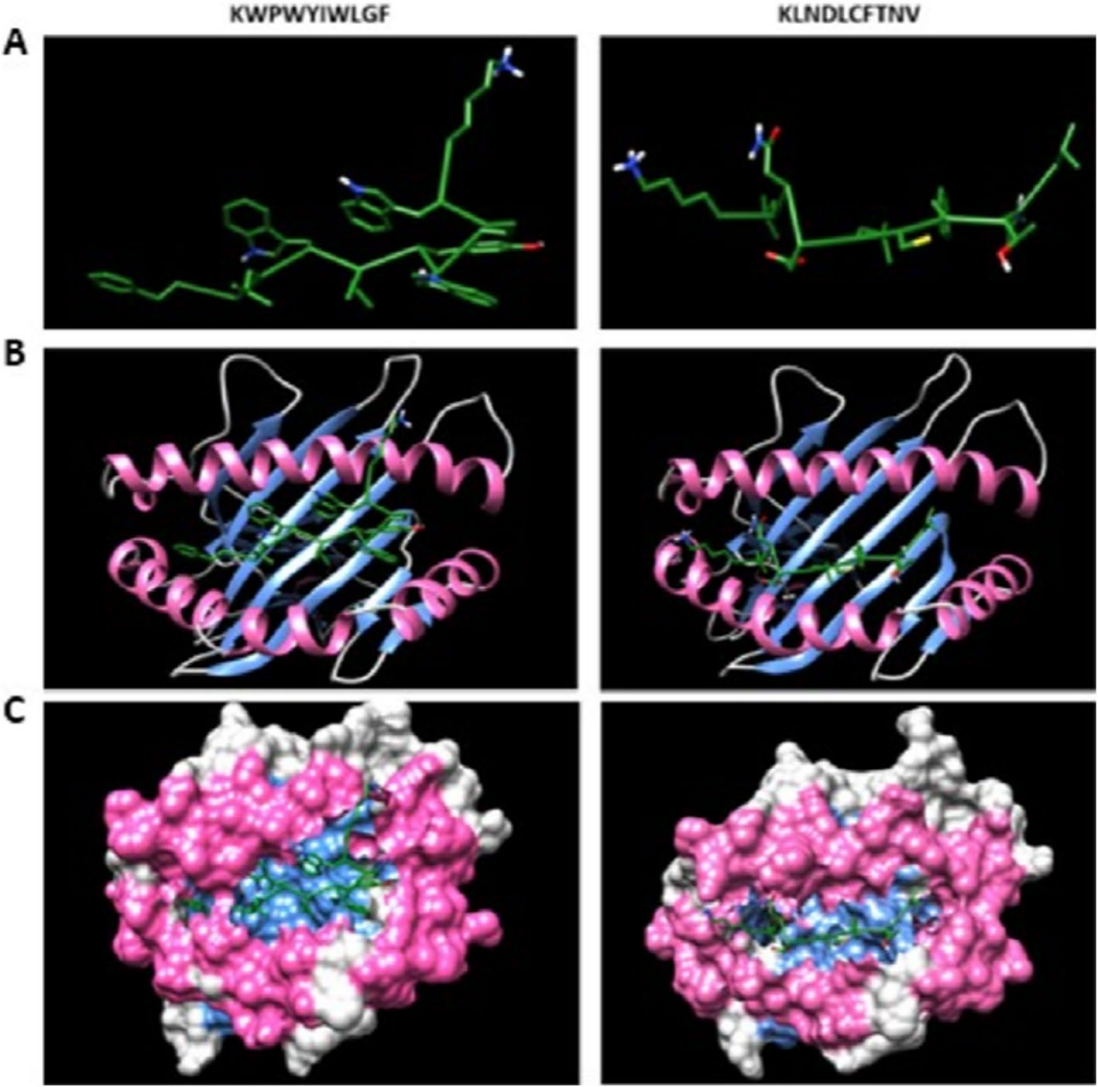
(**A**) Predicted KWPWYIWLGF and KLNDLCFTNV epitopes docking to MHC-I alleles. (**B**) Docking results of epitopes with α chain of MHC-I alleles using ClusPro. (**C**) The snapshot representing the epitope docked in the pocket of molecular surface of the receptor (all the structures are visualised using Chimera 1.14).

**Figure 2:**
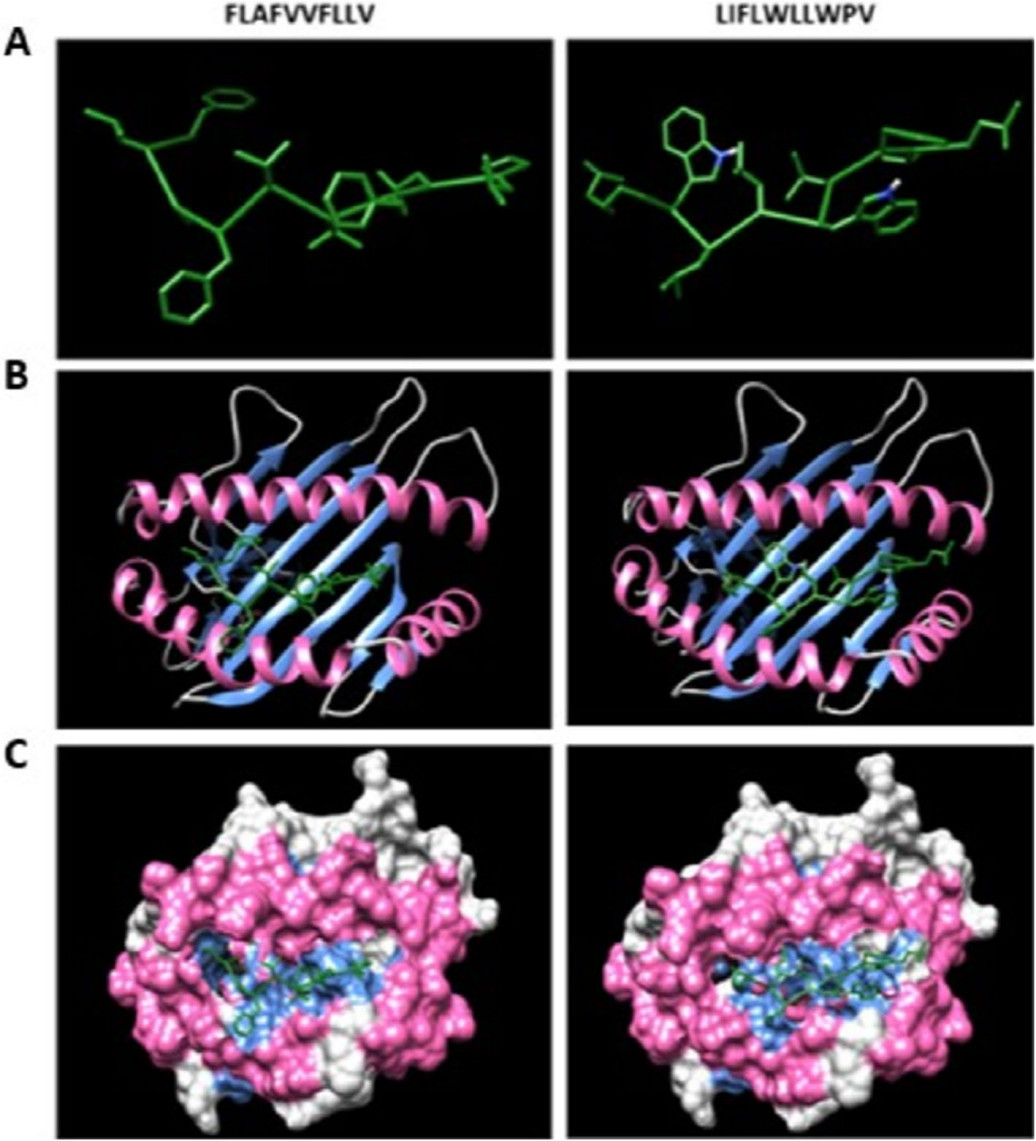
(**A**) Predicted FLAFVVFLLV and LIFLWLLWPV epitopes docking to MHC-I alleles. (**B**) Docking results of epitopes with α chain of MHC-I alleles using ClusPro. (**C**) The snapshot representing the epitope docked in the pocket of molecular surface of the receptor (all the structures are visualised using Chimera 1.14).

**Figure 3:**
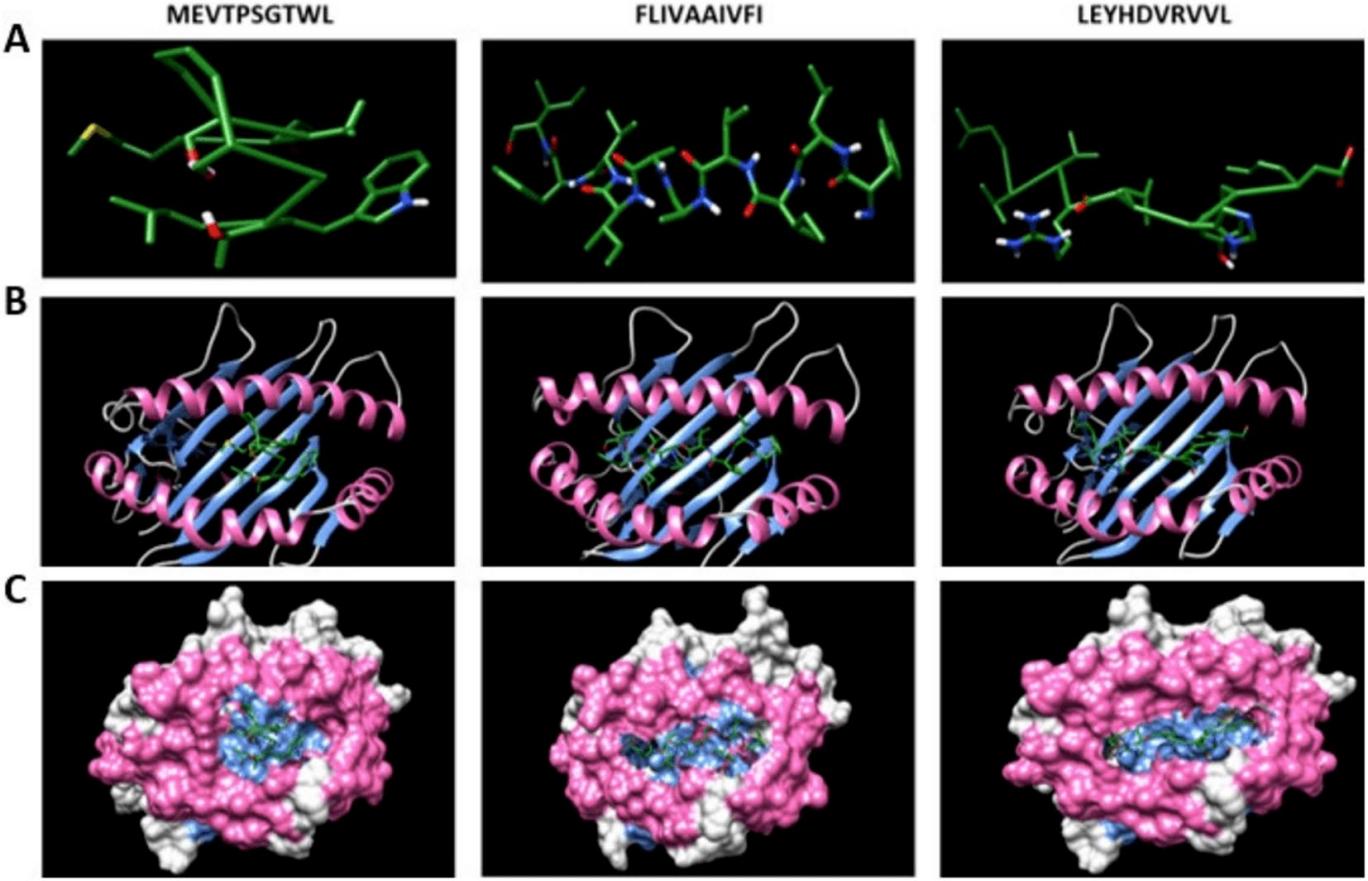
(**A**) Predicted MEVTPSGTWL, FLIVAAIVFI and LEYHDVRVVL epitopes docking to MHC-I alleles. (**B**) Docking results of epitopes with α chain of MHC-I alleles using ClusPro. (**C**) The snapshot representing the epitope docked in the pocket of molecular surface of the receptor (all the structures are visualised using Chimera 1.14).

All probable antigenic epitopes that have a low IC50 value and percentile rank could not be docked with their MHC-I or MHC-II alleles because of limitations associated with available MHC-I and MHC-II alleles variations in data bank or server. Accordingly, KWPWYIWLGF, KLNDLCFTNV, FLAFVVFLLV, LIFLWLLWPV, MEVTPSGTWL, FLIVAAIVFI, and LEYHDVRVVL epitopes belonging to S (including variant D614G), E (including variant L37H), M (including variant T175M, N (including variants P13L, S194L, S197L, and R203K/G204R), orf7a and orf8 (including variants S24L, V62L, and L84S), respectively, were docked with receptors of selected MHC-I alleles (Figs. 1, 2, and 3).

During docking analysis conducted by MHC-II alleles, in S protein, core regions of PTNFTISVTTEILPV, SIIAYTMSLGAENSV, and GYFKIYSKHTPINLV epitopes were docked with the receptor of HLA-DRB1*07:01. Also, the core region of another epitope (QDLFLPFFSNVTWFH) in S protein was docked with the receptor of HLA-DRB1*15:01. In M protein, core regions of ASFRLFARTRSMWSF, RTLSYYKLGASQRVA and PKEITVATSRTLSYY epitopes were docked with the receptor of HLA-DRB1*07:01. Also, the core region of an epitope (QIAQFAPSASAFFGM) in N protein was docked with the receptor of HLA-DRB1*07:01. Similarly, the core region of an epitope (VTLAILTAHRLCAYC) in variant L37H was docking to the receptor of HLA-DRB1*1501. These epitopes that were docked with own MHC-II alleles were also available in their variant proteins.

### Solvent-exposure positions in epitopes

The amino acids exposed to the solvent were detected in all significant epitopes. Among these epitopes, the whole of only two epitopes (FLAFVVFLLV and KWPWYIWLGF) were in the solvent accessible region on protein structure (Table 9).

## Discussion

Reverse vaccinology plays an important role in the development of recombinant vaccines by allowing in silico analyses of the genome of pathogens. In silico analyses enables identifying the highly antigenic and secreted proteins which are crucial in vaccine development before the beginning of the wet lab studies^8,14^. Using this approach, the present study aimed to discover likely antigenic proteins as well as epitope regions that are targeted by both B and T cell arms of the adaptive immune response for the development of a vaccine or serodiagnostic assay as described by Dangi et al.^8^ and Goodswen et al.^14^.

All proteins of SARS-CoV-2, except nsp-16 encoded by orf1ab, were predicted as probable antigen. Although, there was no major difference between predicted antigenicity values for probable vaccine candidate proteins, S protein was selected as a better vaccine candidate protein compared to others depending on in silico analyses results. The physico-chemical analysis showed that S protein had a negative GRAVY value indicating that S protein is hydrophilic and has a better interaction with surrounding water molecules^15^. Also, it had stable and soluble characteristics which are important parameters for biophysical studies on epitope-based vaccine design. Moreover, S protein had a moderate aliphatic index which indicates stability in a wide spectrum of temperature^16^, fewer than two transmembrane helices facilitating cloning, expression, and purification^11^, and a big molecular weight and long estimated half-life (more than 10 h). These properties show that S protein can be used as a vaccine candidate antigen. In addition to these physico-chemical properties, other predicted parameters such as the presence of a signal peptide that increase the immune response and the presence of betawrap motifs that are a virulence factor, as well as a non-allergic property also showed that S protein was a better vaccine candidate. In addition, orf7a, orf8 and nsp-10 proteins were predicted to have a signal peptide. This feature is an important parameter which indicates that the protein can be destined towards the secretory pathway^17,18^. Moreover, the signal peptide promotes protein secretion, and thus, the signal peptide is used to improve the protein secretion level in recombinant techniques^19^. For example, a study showed that vaccination with an unconventionally secreted viral nonstructural protein (NS1) protected mouse from murine norovirus^20^. Also, it has been reported that proteins with the signal peptide should be taken into consideration as vaccine candidates both they have been targeted to the secretory pathway and have high antigenicity and specificity^21^. Accordingly, these probable secreted and antigenic three proteins (orf7a, orf8, and nsp-10) can also be considered as potential vaccine candidate proteins. As S, orf7a, orf8, and nsp-10 proteins examined with regard to secondary structure, random coils were detected higher than 49%. The presence of this highly predicted random coil shows that these proteins can be preferably recognised by an antibody^22^. Another critical point for these proteins was the prediction of post-translational modifications. The presence of these modifications indicates that if these proteins are produced by recombinant technology, eukaryotic expression systems such as yeast, insect or mammalian should be preferred instead of bacterial systems^23^.

In previous vaccine studies, S and M proteins have been used for the development of DNA or recombinant protein vaccines against SARS-CoV that affected 30 countries in five continents^24,25^. Also, S protein has been used to develop a vaccine against MERS CoV which is another zoonotic pathogen that has infected approximately 2500 people in over 25 countries^7,26^. According to the results obtained from these studies, S and M proteins were reported to induce a strong immune response. For the vaccine development against SARS-CoV-2, it has been stated that S protein is a promising candidate because it plays role in viral attachment, fusion, and entry^27–29^. In addition, a report showing that antibodies against S protein of SARS-CoV inhibit the SARS-CoV-2 S protein-mediated entry into cells encourages the use of this molecular target for vaccination^28,29^. Currently, a lot of companies or research groups target the S protein to develop a vaccine against SARS-CoV-2 using various recombinant vaccine technologies. For example, Inovio using S protein with a DNA vaccine technology is in Phase I. Another company, Moderna, is in Phase I/II with an RNA based vaccine targeting S protein^30^. Consequently, these findings of recent studies and our in silico study support that only S proteins can be a strong vaccine candidate protein in the development of a recombinant vaccine against SARS-CoV-2 causing COVID-19.

Since N protein does not locate at the surface of SARS-CoV-2, it was thought that N protein may not be a proper vaccine candidate but could be a good antigen for serodiagnosis of COVID-19 because of having a negative GRAVY value and soluble characteristics and not transmembrane helices. There were several studies for the previous coronavirus (SARS-CoV) supporting our predictions. For example, a previous study reported a strong antibody response against recombinant N protein in 10 of 12 SARS patients^31^. In a different study, a B cell epitope region between 156 and 175 positions of N protein reacted strongly with sera from SARS patients^32^.

Among structural proteins, subcellular localisations of S and E proteins were predicted as endoplasmic reticulum using in silico methods in the current study, and this result was found to be compatible with the results of SARS-CoV performed with an in vitro immunofluorescent analysis showing the localisation of S protein in several compartments of host secretory pathway from the endoplasmic reticulum to cell membrane as well as E protein in endoplasmic reticulum^33^. However, subcellular localisation of M protein was predicted as the host cell membrane and endoplasmic reticulum in the current in silico analysis while it was shown in the Golgi apparatus in the same in vitro analysis^33^. In fact it was also thought to be compatible with in silico results because endoplasmic reticulum, Golgi, and cell membrane are parts of the same host secretory pathway and all surface proteins may be detected in each part of the pathway.

In this study, the immunological effects of prevalent variant proteins belonging to E, M, N, S, and orf8 proteins were also analysed. Accordingly, the comparison of reference S protein and its variant (D614G) showed no difference in antigenicity values, epitope regions, and antigenicity values of epitopes. However, detecting D614G variation as prevalent has been associated with selection advantage and random founder effect^34,35^. In addition to these, a study reported that the D614G variant was more stable and enhanced its infectious nature^36^ whereas another study reported that there was not enough evidence to express that the variant is more infectious^37^. For N protein, among five variations (P13L, S194L, S197L, R203K/G204R), P13L and S197L variations were predicted to increase the antigenicity value of N protein and thus, utilise of P13L and S197L variants was thought to be a better antigen for studies conducted in countries harboring SARS-CoV-2 isolates with P13L or S197L variant. A similar result was also detected in E protein and a higher antigenicity value was predicted in variant L37H. Variant orf8 (L84S) had a lower antigenicity value whereas a higher antigenicity value was predicted in variant orf8 (V62L) compared to orf8 of Wuhan isolate. Also, variant M protein (T175M) had a lower antigenicity value. As depending on these results, since a higher antigenicity value is associated with a stronger immune response in the host, selection of the proteins with high antigenicity values in vaccinological or serodiagnostic studies would be useful.

In the second part of our study, epitope regions specific to B and T cells were predicted in all structural proteins, variants of structural proteins, and non-structural proteins that have a signal peptide, and antigenicity control was performed for all predicted epitopes. Results associated with B cell epitopes showed that there were a lot of highly antigenic epitopes. Antigenicity value was very high for GGDGKMKD, THTGTGQ, and NLDSKV epitopes corresponding to N, nsp-10 encoded by orf1ab and S proteins. Similarly, epitopes that have high antigenicity values were also predicted for MHC-I and II alleles. Among these predicted epitopes, for MHC-I alleles, KLNDLCFTNV (Fig. 1) and ITLCFTLKRK epitopes belonging to S and orf7a proteins had very high antigenicity values whereas for MHC-II alleles, PTNFTISVTTEILPV and IVFITLCFTLKRKTE epitopes belonging to S and orf7a proteins also had significant antigenicity values.

These findings indicate that a cocktail/mixture composed of these epitopes may induce a neutralising antibody response or can be used in the development of an epitope-based peptide vaccine because of their association with both B and T cells. Also, it was thought that they can be used as antigens that capture IgM and IgG antibodies against SARS-CoV-2 during viral infection in ELISA or Western blotting tests. In previous wet lab studies, the presence of neutralising epitopes has been reported to bind with S protein of SARS-CoV^38–40^. For example, in a study conducted in mice, a major neutralisation determinant was reported in receptor-binding domain (RBD) of S protein in SARS-CoV^38^. Another study reported that the epitope NYNWKR in S protein had a neutralising effect against SARS-CoV^39^. There are also some new studies using wet lab techniques and in silico approaches associated with SARS-CoV-2. In a study, splenocytes were stimulated with plenty of T cell epitopes belonging to S protein, and nine of them were reported to induce a cellular immune response. Among these epitopes, only one of them (VGGNYNYLYRLFRKS; between 445 and 459 positions) was inside RBD, five of them (YNYKLPDDFTGCVIA; DDFTGCVIAWNSNNL; VVLSFELLHAPATVC; LLHAPATVCGPKKST; KNKCVNFNFNGLTGT) were located nearby RBD whereas the remaining three (SFPQSAPHGVVFLHV; PHGVVFLHVTYVPAQ; FTTAPAICHDGKAHF) were inside S2 segment of S protein^41^. Interestingly, in our study, an epitope (CYFPLQSYGF; between 488 and 497 positions) with a relatively lower antigenicity value was predicted in RBD of S protein and four epitopes (NLDSKV, KLNDLCFTNV, RQIAPGQTGK, GDEVRQ) were also predicted in a very close region. Docking results supported that the epitope KLNDLCFTNV was targeted by HLA-A*02:01 allele (Fig. 1). These findings indicate that the above-mentioned epitopes may have a promising neutralising effect against SARS-CoV-2. In a previous in silico study, five different epitopes (SYGFQPTNGVGYQPY; SQSIIAYTMSLGAEN; IPTNFTISVTTEILP; AAAYYVGYLQPRTFL; APHGVVFLHVTYVPA) related to both MHC-I and II were predicted in S protein^42^ and only one of them overlapped with a highly antigenic epitope (PTNFTISVTTEILPV) predicted in our study. In another in silico study, 14 epitopes were predicted in S protein for T cells^43^ and six of them were detected to overlap with epitopes predicted in our study. However, none of these overlapped epitopes were among the significant epitopes identified in this study.

Docking analysis confirming the interaction among predicted epitopes and MHC-I/II alleles allows the prediction of more reliable epitopes that can be used for wet lab studies. In this study, docking analysis could not be performed for each of the all MHC-I or MHC-II epitopes due to the lack of 3D structures of some MHC-I/II alleles in protein database or server. This situation limits the docking analysis part of this study as preventing the analysis of all epitopes. Therefore, it was thought that increasing the number of 3D models of MHC-I or II alleles in PDB or servers would be useful for the analysis of a more robust epitope.

Development of computer-based methodologies enhances the credibility of in silico approaches in biological studies. Based on that, the methods of predicting vaccine candidate proteins are always favored even though they are not expressed in vitro. In silico methods also have the advantage of being able to make a fast and cost-efficient analysis. The other advantage of these methods is that they make predictions depending on the structure of vaccine candidate proteins and constitute a major way for vaccine design. Thus, in silico methods can be used at a very early stage in the vaccine development process and this makes in silico methods essential as a pre-analysis approach before starting wet lab studies. Although the multiple numbers of proteins can be analysed by in silico methods for vaccine design studies, there are some limitations that should be taken into consideration. The lack of information in databases, inaccuracies of software algorithms, and usage of inappropriate tools for data are known limitations in terms of in silico studies. Therefore, it is important to select the right tools for analysis and utilize different parameters to find the correct results for in silico-based studies.

## Conclusion

As spike protein has important roles such as viral attachment, fusion and, entry, it is a very significant strategic target for vaccine studies and a lot of companies and research groups use the protein for vaccine development. Our reverse vaccinology in silico approach also supports that S protein is the best vaccine candidate protein. In addition, probable secreted orf7a, orf8, and nsp-10 proteins with signal peptide can be promising vaccine candidates. Epitopes predicted in S protein and other proteins having a signal peptide may have a potential neutralising effect and can be used to develop an epitope-based peptide vaccine or a serodiagnostic assay. In the future, in addition to the currently studied S protein, antigenicity of orf7a, orf8, and nsp-10 proteins as well as significant epitopes selected in this study should be checked by wet lab studies and antigenic proteins/epitopes should be studied as vaccine or serodiagnostic candidates.

## Methods

### SARS-CoV-2 Wuhan isolate and variant proteins

NCBI (National Center for Biotechnology Information) (https://www.ncbi.nlm.nih.gov) was used to obtain the full genome of SARS-CoV-2 isolate (reference isolate Wuhan-Hu-1; Accession number: NC_045512.2 and sequenced in December 2019) and alignment and editing of the full genome was performed by MEGA 7 and BioEdit (Version 7.2)^44,45^. In addition to this reference genome, according to Nextstrain results showing the prevalent variations, variant D614G corresponding for S protein, variant T175M corresponding for M protein, variants P13L, S194L, S197L, R203K, and G204R corresponding for N protein, and variants S24L, V62L, and L84S corresponding for orf8 were selected as additional proteins to investigate the immunological effects associated with antigenicity, signal peptide, BetaWrap motifs, PTMs and, epitope regions. Variants were selected for only structural proteins and the proteins that were predicted to have a signal peptide.

### Prediction of physico-chemical parameters and secondary structures

The reference genome proteins were investigated using Expasy ProtParam online server (https://web.expasy.org/protparam/) for the prediction of physico-chemical properties^46^. The prediction of solubility was performed by SolPro (http://scratch.proteomics.ics.uci.edu/)^47^. Also, prediction of secondary structures was performed by GOR IV online server (https://npsa-prabi.ibcp.fr/cgi-bin/npsa_automat.pl?page=/NPSA/npsa_gor4.html)^48^.

### Prediction of antigenicity

The reference genome proteins as well as variant proteins and predicted epitopes were analysed by Vaxijen v2.0 online server (http://www.ddg-pharmfac.net/vaxijen/VaxiJen/VaxiJen.html) for the prediction of antigenicity using a threshold value of 0.4^49^.

### Prediction of subcellular localisation and number of transmembrane helices

The subcellular localisation of virus proteins in infected host cells were predicted by Virus-mPLoc (http://www.csbio.sjtu.edu.cn/bioinf/virus-multi/)^50^. For the prediction of the number of transmembrane helices, TMHMM Server v. 2.0 (http://www.cbs.dtu.dk/services/TMHMM/) was used^51^.

### Prediction of signal peptide

The reference genome proteins and variant proteins were analysed by Signal-BLAST (http://sigpep.services.came.sbg.ac.at/signalblast.html)^52^.

### Prediction of allergenicity

The allergenicity of the reference genome structural proteins, variant proteins, and the proteins that have a signal peptide was predicted by Algpred online server (http://crdd.osdd.net/raghava/algpred/) using a prediction approach of MEME/MAST motif and IgE epitopes^53^.

### Prediction of BetaWrap motifs

The prediction of BetaWrap motifs of the reference genome structural proteins, variant proteins, and the proteins that have a signal peptide was carried out by BetaWrap online server (http://cb.csail.mit.edu/cb/betawrap/betawrap.html)^54^.

### Prediction of similarity with host proteome

The reference genome structural proteins, variant proteins, and the proteins that have a signal peptide were examined by BlastP (https://blast.ncbi.nlm.nih.gov/Blast.cgi?PAGE=Proteins) to predict the similarity with the host proteome. In analysis, *Homo sapiens* was selected as a host organism.

### Prediction of post-translational modifications

The prediction of post-translational modifications of the reference genome structural proteins, variant proteins, and the proteins that have a signal peptide were carried out using NetNGlyc 1.0 server (http://www.cbs.dtu.dk/services/NetNGlyc/)^55^, NetOGlyc 4.0 server (http://www.cbs.dtu.dk/services/NetOGlyc/)^56^, NetPhos 3.1 server (http://www.cbs.dtu.dk/services/NetPhos/)^57^ and, GPS-MSP and GPS-PAIL running under CSS-Palm Online Service (http://csspalm.biocuckoo.org/online.php)^58^. In addition, NetSurfP 2.0 (http://www.cbs.dtu.dk/services/NetSurfP/) was used for the prediction of surface accessibility of post-translational modification sites in proteins^59^.

### Prediction of B cell epitopes

Linear B cell epitopes of the reference genome structural proteins, variant proteins, and the proteins that have a signal peptide were predicted by Bcepred (http://crdd.osdd.net/raghava/bcepred/)^60^ and Bepipred Linear Epitope Prediction 2.0 running under IEDB (the immune epitope database, https://www.iedb.org/)^61^ online servers.

### Prediction of MHC-I and MHC-II epitopes

The prediction of MHC-I and MHC-II epitopes of the reference genome structural proteins, variant proteins, and the proteins that have a signal peptide were analysed by IEDB (https://www.iedb.org/)^61^. For the prediction of MHC-I epitopes, twelve different MHC-I alleles (A01.01, A02.01, A03.01, A24.02, A26.01, B07.02, B08.01, B27.05, B39.01, B40.01, B58.01 and B15.01) which are HLA super-type representative were utilised in the analysis. For the prediction of MHC-II epitopes, seven different MHC-II alleles (DRB1.03.01, DRB1.07.01, DRB1.15.01, DRB3.01.01, DRB3.02.02, DRB4.01.01 and DRB5.01.01) were used in the analysis.

### Docking analysis with MHC-I and II alleles

For docking analyses conducted with MHC-I alleles, receptor alleles that were specific to each epitope were retrieved from Protein Data Bank (PDB; http://www.rcsb.org/pdb/). In selection of MHC-I receptor models, the presence of free (undocked) 3D protein structures were considered. Models of epitopes that were selected based on low IC50 value and being probable antigen were predicted by I-TASSER Server (http://zhanglab.ccmb.med.umich.edu/I-TASSER)^62^. In addition, epitopes that have the highest antigenicity value were also selected for docking. Each modelled epitope ligand was docked to its specific MHC-I allele receptor by ClusPro Server (https://cluspro.bu.edu/home.php)^63^ and visualised on UCSF Chimera 1.14 tool^64^. For docking analyses conducted with MHC-II alleles, each epitope that was selected based on low IC50 value and being probable antigen was docked to its specific MHC-II allele by selecting specific alleles from the EpiDock Server (http://www.ddg-pharmfac.net/epidock/EpiDockPage.html)^65^.

### Prediction of solvent-exposure positions in epitopes

Each protein was aligned with its variants by Clustal Omega (https://www.ebi.ac.uk/Tools/msa/clustalo/)^66^. Then, a 3D structure pdb file for each protein was downloaded from the Protein Data Bank (PDB; http://www.rcsb.org/pdb/) or constructed by modelling using Swiss-Model (https://swissmodel.expasy.org). As an input for each protein, an alignment file and a 3D protein model file were uploaded and run on ESPript 3.0 (http://espript.ibcp.fr/ESPript/ESPript/)^67^ to predict amino acid solvent-exposure (accessibility) properties for epitopes.

### Comparison of significant epitopes among major lineages

For representation of each of 11 major lineage (including A, A.1, A.2, A.3, A.5, B, B.1, B.1.1, B.2 B.3, B.4) given in a study^68^, 11 SARS-CoV-2 genome sequences were retrieved from GISAID database (https://www.gisaid.org) and checked for lineage analysis using Pangolin (https://pangolin.cog-uk.io). Then, protein coding regions for S, M, N, E, orf7a, orf8, and nsp-10 were translated and compared to find epitope differences among 11 SARS-CoV-2 sequences representing major lineages. Accession numbers for major lineages are MT049951, EPI_ISL_420879, LC528233, EPI_ISL_416538, EPI_ISL_530117, MT020781, EPI_ISL_420910, EPI_ISL_418263, MT039890, EPI_ISL_529598, NC_045512.
